# Probability-Driven Adaptive Foot-Mounted Inertial Navigation with Local Straight-Heading and Stair-Height Constraints

**DOI:** 10.3390/s26185967

**Published:** 2026-09-21

**Authors:** Dongpeng Xie, Peihui Yan, Yifei Li, Qinghai Wang, Jingnan Liu

**Affiliations:** 1 GNSS Research Center, Wuhan University, Wuhan 430079, China; dongpengxie@whu.edu.cn (D.X.); yfeili@whu.edu.cn (Y.L.); jnliu@whu.edu.cn (J.L.); 2 Hubei Luojia Laboratory, Wuhan 430079, China; wangqinghai@whu.edu.cn; 3 University Student Engineering Training and Innovation Practice Center, Wuhan University, Wuhan 430072, China

**Keywords:** foot-mounted inertial navigation, GNSS-denied navigation, pedestrian dead reckoning, zero-velocity update, gait classification, heading drift, stair-height constraint, LightGBM

## Abstract

Foot-mounted inertial pedestrian navigation is an attractive infrastructure-free solution for pedestrian positioning in environments where Global Navigation Satellite System (GNSS) signals are unavailable, degraded, or intentionally excluded. However, mixed-gait and multi-level motion expose two persistent failure modes: unreliable zero-velocity detection during high-dynamic motion and weak yaw observability after standard zero-velocity-aided filtering. This paper presents a probability-driven adaptive foot-mounted inertial navigation framework that couples a lightweight motion-probability front-end with probability-weighted mode-adaptive zero-velocity detection, local straight-heading constraints, and probability-gated stair-height updates. A 335-dimensional motion-feature LightGBM classifier first outputs six gait probabilities from 128-sample windows of six-axis inertial data. These probabilities continuously determine the zero-velocity detector threshold and the reliability of heading and vertical pseudo-measurements. For planar navigation, a motion-aware local straight-heading constraint (MA-LSHC) establishes local reference headings only over curvature-safe straight segments, and an adaptive heading covariance controls the strength of each heading pseudo-measurement. For stair navigation, height changes are quantized into integer-riser increments only when stair probabilities and vertical inertial evidence agree. All data were collected in outdoor pedestrian environments, and the evaluation includes four parts: motion classification, training and detector diagnostics, closed rectangular planar navigation, and multi-level stair-height reconstruction. The motion-feature classifier achieves 96.24% accuracy and 96.17% macro-F1. In a mixed-gait closed rectangular route, the proposed method obtains a 1.09 m closure error and a 0.68% relative closure error, outperforming fixed SHOE-ESKF, Wang-AWGF, Guo-SIHDC, and Deng-HDR baselines. In stair experiments, the proposed method achieves a grid-node RMSE of 0.13 m and correctly identifies 38 ascending and 38 descending risers. These results demonstrate that motion probabilities can be used not merely as labels, but as continuous reliability cues for adaptive inertial constraints.

## 1. Introduction

Foot-mounted inertial navigation is a self-contained pedestrian positioning technique in which a shoe-mounted inertial measurement unit (IMU) is propagated by strapdown mechanization and repeated stance phases provide zero-velocity pseudo-measurements. During each detected stance, a zero-velocity update (ZUPT) constrains the accumulated velocity error and improves roll/pitch and inertial-bias estimates [1,2,3,4]. Because this architecture does not require pre-installed infrastructure, it remains attractive when GNSS, wireless positioning, or visual landmarks are unavailable or unreliable [5,6,7,8]. Its principal limitations arise when the assumptions behind these pseudo-measurements become unreliable. Zero-velocity intervals are less distinct during running, stair motion, or irregular gait, while yaw remains weakly observable under a ZUPT alone; consequently, small heading errors can produce substantial lateral position drift over long straight segments or closed-loop routes [9,10,11].

Although foot-mounted inertial navigation is frequently discussed in the context of indoor positioning, its underlying value is broader: it provides a self-contained positioning solution wherever GNSS is unavailable, degraded, or deliberately not used. In this study, all physical experiments were conducted in controlled outdoor pedestrian environments. The navigation estimator used only foot-mounted inertial measurements; scene geometry, marked routes, and reference points were used solely for documentation and performance evaluation. This experimental design provides observable reference structures without changing the GNSS-independent nature of the proposed method.

Recent work has improved different pieces of this pipeline. Adaptive zero-velocity detection adjusts test thresholds, learns stance decisions from inertial sequences, or maps contact probabilities to measurement uncertainty [12,13,14]. Motion-classification-aided navigation uses gait mode information to switch filters, lock height, or update motion constraints [15,16]. Heading-drift reduction methods introduce heuristic drift reduction, zero-integrated-heading-rate constraints, or interval heading-difference corrections [5,16,17]. However, many existing solutions still use motion state mainly as a hard label, and the resulting switches can be brittle near gait transitions. A probability representation is more informative because it encodes uncertainty and permits smooth changes between gait regimes. For an error-state Kalman filter (ESKF), the key issue is therefore not only whether the activity classifier is correct, but also how classification uncertainty is translated into observation generation and observation covariance. In the present framework, the motion classifier does not replace the physical state estimator; instead, its probability vector acts as a reliability interface that continuously regulates the pseudo-measurements used by the ESKF.

This paper develops a probability-driven adaptive foot-mounted inertial navigation framework. Instead of designing a complex deep network as the central claim, we use a compact multi-domain feature classifier to produce reliable motion probabilities. These probabilities then become continuous reliability cues for the navigation filter. The planar component suppresses heading drift with a local straight-heading pseudo-measurement whose covariance is controlled by straightness, gait suitability, and classification confidence. The vertical component applies a stair-height update only when stair probabilities and inertial height increments support the same decision.

The main contributions of this work are summarized as follows:

- A lightweight motion-probability front-end is constructed using time-domain, frequency-domain, correlation, and periodicity features. It outputs six-mode probabilities and achieves 96.24% accuracy under a held-out-subject protocol with per-subject unlabeled normalization.
- A probability-driven mode-adaptive SHOE detector and MA-LSHC heading constraint are proposed. The zero-velocity threshold is continuously mixed from six gait probabilities, while the heading pseudo-measurement covariance is adjusted using local straightness, gait suitability, and classifier confidence.
- A probability-gated integer-riser height update is designed for multi-level motion. It activates the riser-height constraint only when the stair probability and vertical inertial evidence are consistent, thereby reducing vertical drift while avoiding hard-label gate failures.

The rest of this paper is organized as follows. Section 2 reviews recent foot-mounted navigation and wearable motion-recognition studies. Section 3 presents the probability front-end, adaptive zero-velocity detector, MA-LSHC, and stair-height update. Section 4 reports the four experimental parts. Section 5 discusses limitations and reproducibility, and Section 6 concludes this paper.

## 2. Related Work

### 2.1. Foot-Mounted Inertial Navigation and Adaptive Zero-Velocity Updates

Classical foot-mounted navigation uses the repeated stance phase of the foot as a zero-velocity observation. Early and representative implementations demonstrated this principle using boot- or shoe-mounted inertial units, sometimes supplemented by pressure or GNSS measurements for evaluation or aiding [1,6,7,8]. A Kalman filter can then correct velocity, roll/pitch error, and inertial-sensor biases [2,3,4]. The limitation is directional observability: a ZUPT does not directly observe yaw, so small heading errors may become large cross-track errors on long corridors or closed loops [5,10,11].

A fixed SHOE threshold is also unreliable across static, walking, running, and stair motion. Learned sequence detectors have been introduced to reduce gait- and user-dependent threshold tuning [14]. Wang et al. used gait-frequency information to adapt the detector window and threshold [12], while Gao et al. represented contact by a posterior probability and converted it into an effective velocity-measurement covariance [13]. These methods improve contact detection, but their observation is still velocity. They are therefore suitable adaptive-ZUPT references, not complete solutions to yaw drift. The present work also uses a continuous representation, but the same probability vector controls ZUPT, heading, and height observations rather than only contact.

### 2.2. Heading Constraints and Motion-Aware Drift Reduction

Heuristic heading-drift reduction exploits periods of approximately straight walking to estimate or suppress slowly varying gyro drift [18,19]. Related constraint-based foot-mounted systems introduced building-heading, zero-integrated-heading-rate, and height updates to improve otherwise weak directional and vertical observability [20]. HDR assumes that pedestrians often move locally straight and suppresses small step-to-step heading changes [5]. ZIHR relates integrated heading change during stationary intervals to gyroscope bias. Deng et al. combined ZIHR and simplified HDR in a gait-classification-aided 3D system [16]; Guo et al. later proposed SIHDC, which uses motion recognition to support interval heading-difference correction [17]. Both show that a relative heading-change constraint can outperform velocity-only constraints, although the global yaw offset remains unobservable without an external direction reference.

The remaining issue is reliability. Fixed straightness thresholds may reject useful segments or over-constrain gradual turns, and interval corrections may be sensitive to gait transitions. MA-LSHC therefore uses a local circular-mean reference heading, rejects persistent curvature, and maps straightness, gait suitability, and classifier entropy to the observation covariance. It does not use known corridor directions, ninety-degree snapping, opposite-side equality, or loop closure.

### 2.3. Motion Classification and Lightweight Wearable Models

Wearable human-activity recognition has been organized around feature extraction, segmentation, sensor placement, and evaluation protocols in several surveys and tutorials [21,22]. Deep sequence models have subsequently been studied across representative wearable datasets [23,24]. DeepConvLSTM is a common wearable baseline because it combines temporal convolution with recurrent context [25]. Recent lightweight models use sensor-wise attention, compact transformers, depthwise convolution, or state-space layers [26,27,28,29,30]. For navigation, however, probability quality is as important as hard-label accuracy because overconfident errors can create overly strong pseudo-measurements. The Brier score is a classical proper score for probabilistic forecasts [31], and modern classifiers can require explicit calibration analysis even when their hard-label accuracy is high [32]. This motivates reporting the NLL and Brier score together with F1.

The selected LightGBM front-end uses 335 explicit features describing amplitude, periodicity, spectral energy, entropy, and cross-axis coupling. Gradient-boosted trees can exploit these gait signatures with fewer trainable degrees of freedom than deep sequence models [33,34]. Learned inertial odometry methods such as RoNIN and TLIO estimate velocity or displacement directly [35,36]; they are not direct baselines here because they require different supervision and output definitions. Our objective is to preserve a physical INS/ESKF and use learning only for reliability estimation.

### 2.4. Multi-Level Height Reconstruction

The vertical position is vulnerable to double integration of acceleration error. Barometers can stabilize altitude but introduce pressure drift and airflow sensitivity [37]. Stair-height constraints instead exploit approximately constant risers [16]. Recent work includes impact-aware foot reconstruction [38], switching vertical constraints [15], and activity-dependent zero-height-change constraints [39]. These approaches improve local height estimation, but they do not all enforce integer-riser consistency over a complete ascent–descent route.

The proposed update combines continuous stair probability with an integer-lattice residual test. Probability gating reduces false activation during level motion, while the lattice test rejects height increments that are incompatible with the estimated riser. This targets both local false updates and long-run vertical drift.

### 2.5. Synthesis and Research Gap

Existing systems differ in the state component they observe, how they represent motion uncertainty, and what environmental assumptions they require. The adaptive ZUPT mainly observes velocity, heading methods add yaw information, and stair methods add vertical geometry. Most use motion recognition as a hard switch and design these observations independently. This work instead uses one six-mode probability vector as a common reliability interface for all three constraints. The experiments consequently evaluate probability quality, planar yaw-drift suppression, and stair-height consistency as connected parts of one estimator.

## 3. Materials and Methods

### 3.1. System Overview

**Figure 1:**
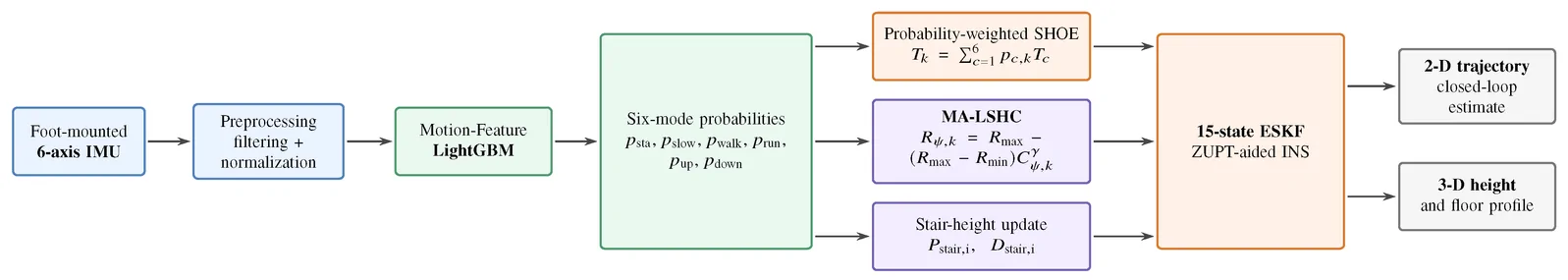
Overall probability-driven adaptive foot-mounted inertial navigation framework. The probabilistic front-end supplies reliability cues to the physical pseudo-measurements; it does not overwrite the ESKF navigation state.

Figure 1 shows the proposed framework. A single six-axis foot-mounted IMU provides synchronized acceleration and angular-rate samples. The processing chain consists of a probabilistic front-end and a physics-based navigation back-end. The front-end operates on overlapping windows, extracts multi-domain motion features, and outputs probabilities for static, slow walking, walking, running, upstairs, and downstairs. In the reported offline evaluation, a window output is associated with its center time. A causal implementation must either delay the output by half a window or time-stamp it at the end of the completed window; only probabilities available at sample time are used by the navigation filter.

The physics-based navigation back-end contains strapdown mechanization and a 15-state error-state Kalman filter. Motion probabilities affect three parts of this navigation back-end. First, they mix six class-specific SHOE thresholds, so contact detection changes smoothly with gait. Second, they regulate the reliability of the MA-LSHC heading observation together with local yaw-rate evidence. Third, they gate the integer-riser update in the vertical channel. The classifier never overwrites the position, attitude, or height. It only changes the acceptance and covariance of physical pseudo-measurements, which limits the effect of an isolated classification error. The system uses no magnetic heading, camera, map, known endpoint, or rectangular geometry.

### 3.2. Motion-Feature Probability Front-End

Let the IMU window be

(1) $$X_{j}=\left[a_{j},\omega_{j}\right]\in R^{L\times 6},\;L=128,$$

where a and ω are the accelerometer and gyroscope measurements. For each window, time-domain statistics, magnitude features, differential statistics, spectral-energy ratios, spectral entropy, dominant frequency, auto-correlation, and cross-channel correlations are concatenated into

(2) $$f_{j}=\Phi \left(X_{j}\right)\in R^{335}.$$

The feature extractor is deterministic and identical for training, validation, and evaluation. Specifically, it first computes channel-wise time-domain descriptors (including the mean, standard deviation, RMS, and differential statistics), then forms acceleration- and angular-rate magnitude descriptors. Frequency-domain processing supplies spectral-energy ratios, spectral centroid, spectral entropy, and dominant frequency. Lagged auto-correlation terms describe periodicity, while pairwise cross-channel correlations describe inter-axis coupling. These quantities are concatenated in a fixed order to produce the 335-dimensional vector in Equation (2); no navigation-state estimate or route geometry is used during feature construction.

A LightGBM classifier (LightGBM Python package; official website: LightGBM documentation: https://lightgbm.readthedocs.io/; accessed on 16 September 2026) then estimates a six-dimensional probability vector

(3) $$p_{j}={\left[p_{\mathrm{sta}},p_{\mathrm{slow}},p_{\mathrm{walk}},p_{\mathrm{run}},p_{\mathrm{up}},p_{\mathrm{down}}\right]}^{⊤},\;\sum_{c=1}^{6}p_{c,j}=1.$$

Window probabilities are mapped to sample time and smoothed using only available outputs, producing $p_{k}$. The normalized entropy

(4) $$H_{k}=-\frac{1}{\log6}\sum_{c=1}^{6}p_{c,k}\log\left(p_{c,k}+\epsilon \right)$$

measures uncertainty, and the classifier confidence is

(5) $$C_{e,k}=1-H_{k}.$$

The feature families encode amplitude, impact sharpness, cadence, periodicity, spectral entropy, and cross-axis coordination. Their complementarity is useful for separating nearby gait classes that cannot be distinguished from one sensor axis alone.

Let the classifier output be indexed by *j*, and let j(k) denote the latest output available at sample *k* after accounting for the window latency described above. Sample-level probabilities are obtained as

(6) $${\tilde{p}}_{c,k}=\frac{\sum_{\ell =0}^{L_{p}-1}q_{\ell }\;p_{c,j(k)-\ell }}{\sum_{\ell =0}^{L_{p}-1}q_{\ell }},\;q_{\ell }\geq 0,$$

where j(k) is the latest window available at sample *k*, and $q_{\ell }$ are fixed smoothing weights. The notation $p_{c,k}$ below refers to the smoothed probability. Smoothing is applied to probabilities rather than hard labels, preserving gradual transitions between modes.

**Figure 2:**
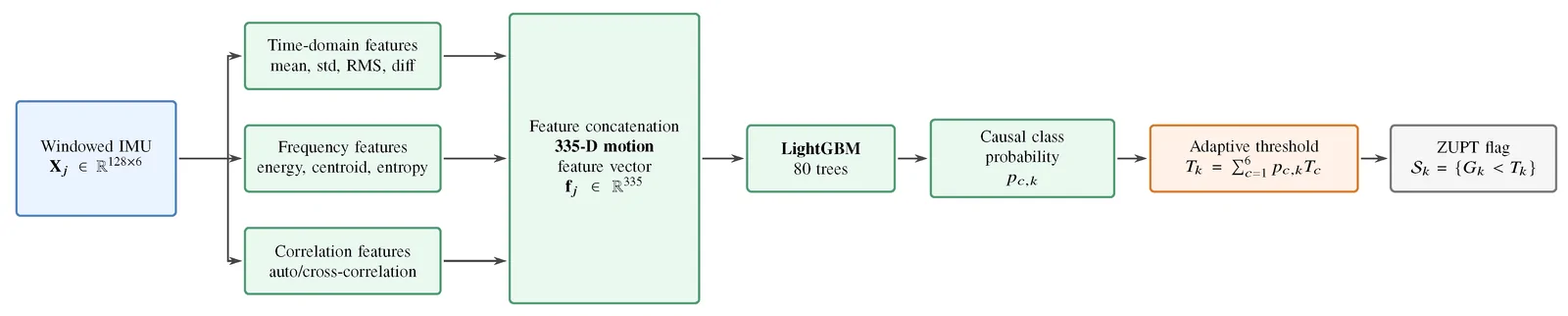
Motion-feature front-end and probability-weighted mode-adaptive SHOE detector. Mathematical notation is consistent with Equations (1)–(9); the displayed class probability denotes the causal probability available to the detector.

Figure 2 summarizes the motion-feature front-end and the probability-weighted SHOE detector.

### 3.3. Probability-Weighted Mode-Adaptive SHOE Detection

For a window $V_{k}$ containing $N_{v}$ IMU samples, the SHOE statistic is written as

(7) $$G_{k}=\frac{1}{N_{v}}\sum_{i\in V_{k}}\left[\frac{1}{\sigma_{a}^{2}}{∥a_{i}-g\frac{{\bar{a}}_{k}}{∥{\bar{a}}_{k}{∥}_{2}}∥}_{2}^{2}+\frac{1}{\sigma_{\omega }^{2}}{∥\omega_{i}∥}_{2}^{2}\right],$$

where ${\bar{a}}_{k}$ is the mean acceleration in the window, and $\sigma_{a}$ and $\sigma_{\omega }$ are detector scaling parameters. A fixed threshold assumes that the contact dynamics are unchanged, which is not valid across static, slow walking, normal walking, running, and stair motion.

The proposed detector uses the probability-weighted threshold

(8) $$T_{k}=\sum_{c=1}^{6}p_{c,k}T_{c},$$

where $T_{c}$ is the frozen threshold calibrated for the *c*th mode. The zero-velocity event is

(9) $$S_{k}=\left\{G_{k}<T_{k}\right\}.$$

Because Equation (8) is a convex mixture, the threshold remains bounded and changes smoothly at gait transitions. The six mode thresholds are calibrated on development data, frozen before evaluation, and are not optimized using the rectangular endpoint.

### 3.4. Error-State Inertial Filter

The nominal state consists of the position, velocity, attitude quaternion, accelerometer bias, and gyroscope bias. Neglecting Earth rotation over the short pedestrian routes, the continuous-time propagation is

(10) $$\begin{array}{cc} \dot{\hat{p}} & =\hat{v}, \end{array}$$

(11) $$\begin{array}{cc} \dot{\hat{v}} & =R\left(\hat{q}\right)\left(a_{m}-{\hat{b}}_{a}\right)+g, \end{array}$$

(12) $$\begin{array}{cc} \dot{\hat{q}} & =\frac{1}{2}\Omega \left(\omega_{m}-{\hat{b}}_{g}\right)\hat{q}, \end{array}$$

(13) $$\begin{array}{cc} {\dot{\hat{b}}}_{a} & =0,\;{\dot{\hat{b}}}_{g}=0, \end{array}$$

where $R(\hat{q})$ rotates body-frame specific force into the navigation frame. Bias instability and sensor noise are represented in the error-state process model.

The 15-dimensional error state is ordered as

(14) $$\delta x={\left[\delta p^{⊤},\delta v^{⊤},\delta \theta^{⊤},\delta b_{a}^{⊤},\delta b_{g}^{⊤}\right]}^{⊤}.$$

The attitude error follows a left-multiplicative, navigation-frame small-angle convention. With $R_{\mathrm{true}}$ and $\hat{R}$ denoting the true and nominal body-to-navigation rotation matrices, respectively,

$$R_{\mathrm{true}}≃\left(I+{\left[\delta \theta \right]}_{\times }\right)\hat{R},$$

where ${\left[\;\cdot \;\right]}_{\times }$ is the skew-symmetric matrix. Equivalently, $q_{\mathrm{true}}≃\delta q⊗\hat{q}$ with $\delta q≃{\left[1,\frac{1}{2}\delta \theta^{⊤}\right]}^{⊤}$. Under this definition, a positive $\delta \theta_{z}$ means that the true yaw exceeds the nominal yaw, which yields the positive yaw-error Jacobian used below.

Its covariance is propagated by

(15) $$\dot{P}=FP+PF^{⊤}+GQG^{⊤},$$

using the standard first-order error dynamics of a strapdown INS [11,40]. The implementation discretizes Equation (15) at the IMU sampling interval.

During a detected stance, the velocity pseudo-measurement is

(16) $$r_{v,k}=0-{\hat{v}}_{k}=H_{v}\delta x_{k}+n_{v,k},\;H_{v}=\left[\begin{array}{ccc} 0_{3\times 3} & I_{3} & 0_{3\times 9} \end{array}\right].$$

The Kalman correction is injected into the nominal state, after which the attitude-error component is reset. The ZUPT provides the common base for all planar methods in the comparison. The proposed heading and height modules add observations without changing the underlying mechanization.

### 3.5. Motion-Aware Local Straight-Heading Constraint

MA-LSHC separates segment safety from measurement strength: curvature gates reject unsafe segments, while confidence controls the covariance of accepted updates.

For a local window $W_{k}$, the yaw-rate descriptors are

(17) $$\begin{array}{cc} \mu_{\omega ,k} & =\left|\frac{1}{|W_{k}|}\sum_{i\in W_{k}}\omega_{z,i}\right|, \end{array}$$

(18) $$\begin{array}{cc} \sigma_{\omega ,k} & =\sqrt{\frac{1}{|W_{k}|-1}\sum_{i\in W_{k}}{\left(\omega_{z,i}-{\bar{\omega }}_{z,k}\right)}^{2}}, \end{array}$$

(19) $$\begin{array}{cc} \Delta \psi_{W,k} & =\left|\sum_{i\in W_{k}}\omega_{z,i}\Delta t\right|. \end{array}$$

A candidate segment must satisfy minimum-duration and curvature gates. Persistent signed yaw rate or excessive accumulated heading change rejects it before any update.

Within an accepted candidate, straightness is quantified continuously as

(20) $$C_{s,k}=\exp\left[-{\left(\frac{\sigma_{\omega ,k}}{s_{\sigma }}\right)}^{2}-{\left(\frac{\mu_{\omega ,k}}{s_{\mu }}\right)}^{2}-{\left(\frac{\Delta \psi_{W,k}}{s_{\psi }}\right)}^{2}\right].$$

The mode-suitability term is

(21) $$C_{m,k}=\sum_{c=1}^{6}\beta_{c}p_{c,k},$$

where $\beta_{c}\in \left[0,1\right]$ expresses how compatible each mode is with a locally constant heading. The classifier-confidence term $C_{e,k}$ is defined by Equation (5). Their product

(22) $$C_{\psi ,k}=C_{s,k}C_{m,k}C_{e,k}$$

ensures that a weak factor cannot be hidden by two strong factors.

The reference heading is a confidence-weighted circular mean over strictly previous accepted stance nodes $I_{k}\subset \left\{i:i<k\right\}$:

(23) $$\psi_{k}^{\mathrm{ref}}=atan2\left(\sum_{i\in I_{k}}w_{i}\sin{\hat{\psi }}_{i},\sum_{i\in I_{k}}w_{i}\cos{\hat{\psi }}_{i}\right),\;w_{i}=C_{\psi ,i}.$$

Circular averaging avoids the discontinuity at ±π, and slow reference adaptation permits small natural heading changes. Because this reference is constructed internally from past estimates, the update constrains local heading change rather than absolute yaw; the global heading gauge remains unobservable.

The relative-heading pseudo-observation is formulated as a zero residual with respect to the local reference. The innovation is

(24) $$r_{\psi ,k}=0-\mathrm{wrap}\left({\hat{\psi }}_{k}-\psi_{k}^{\mathrm{ref}}\right)≃H_{\psi }\delta x_{k}+n_{\psi ,k},$$

with, under a navigation-frame small-angle convention and a near-level stance approximation,

(25) $$H_{\psi }=\left[\begin{array}{ccc} 0_{1\times 6} & e_{3}^{⊤} & 0_{1\times 6} \end{array}\right],\;n_{\psi ,k}∼N\left(0,R_{\psi ,k}\right).$$

The observation directly corrects the yaw-error state and, through cross-covariance, the vertical gyroscope bias. Its covariance is

(26) $$R_{\psi ,k}=R_{\max}-\left(R_{\max}-R_{\min}\right)C_{\psi ,k}^{\gamma }.$$

**Figure 3:**
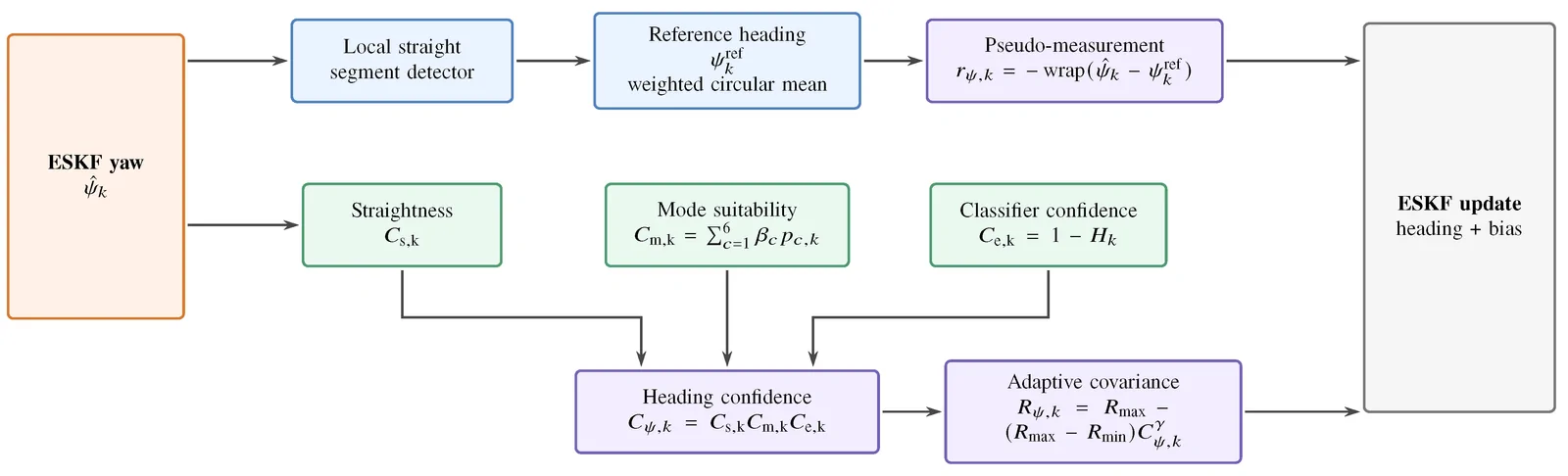
Probability-driven MA-LSHC heading update. Segment safety determines whether an update is allowed, while straightness, mode suitability, and classifier confidence determine the covariance of the accepted relative-heading pseudo-measurement.

A clear straight segment therefore produces a strong correction, whereas an uncertain segment contributes only a weak update. Figure 3 shows the separate yaw and confidence paths.

### 3.6. Probability-Gated Stair-Height Update

The vertical channel is evaluated at stance nodes because velocity is most reliable after a ZUPT. For consecutive stance nodes, the raw vertical increment is

(27) $$\Delta h_{i}=h_{i+1}-h_{i}.$$

Let $K_{i}$ contain the IMU samples between the two stance nodes. The mean stair probability and the signed directional evidence are

(28) $$\begin{array}{cc} P_{\mathrm{stair},i} & =\frac{1}{|K_{i}|}\sum_{k\in K_{i}}\left(p_{\mathrm{up},k}+p_{\mathrm{down},k}\right),\\ D_{\mathrm{stair},i} & =\frac{1}{|K_{i}|}\sum_{k\in K_{i}}\left(p_{\mathrm{up},k}-p_{\mathrm{down},k}\right). \end{array}$$

Using a positive-up height convention, an interval enters the riser-estimation set only when its probability, magnitude, and direction satisfy

(29) $$P_{\mathrm{stair},i}>\tau_{p},\;\tau_{h,\min}<\left|\Delta h_{i}\right|<\tau_{h,\max},\;\Delta h_{i}D_{\mathrm{stair},i}>0.$$

The two-sided magnitude gate removes residual level-walking drift and unrealistically large impulses, while the final condition requires the classifier direction and inertial height change to agree.

Let A={i:thegateinEquation(29)isaccepted} denote the accepted stance-interval index set. The fundamental riser height is estimated from these increments by a robust integer-lattice search: 


(30) r^=argminr∈[rmin,rmax]mediani∈A(minn∈{1,…,nmax}||Δhi|−nr|).

Here, $r_{\min}$ and $r_{\max}$ are the lower and upper physically plausible riser-height bounds, and $n_{\max}$ is the maximum number of risers that one stance interval is allowed to span. These quantities are fixed from the development sequences before evaluation. Restricting $\left[r_{\min},r_{\max}\right]$ is necessary because otherwise integer submultiples can make the fundamental height non-identifiable. The median objective allows one stance interval to span multiple risers while reducing the influence of isolated impact-corrupted increments.

For each accepted interval, the signed integer span is

(31) $$n_{i}=\mathrm{clip}\left(\mathrm{round}\left(\frac{\Delta h_{i}}{\hat{r}}\right),-n_{\max},n_{\max}\right).$$

The update is rejected when the quantization residual $|\Delta h_{i}-n_{i}\hat{r}|$ exceeds the fixed span-dependent tolerance $\tau_{q}\left(\right|n_{i}\left|\right)$, where $\tau_{q}\left(\;\cdot \;\right)$ is calibrated on the development sequences. Otherwise, the corrected stance-node height is

(32) $$h_{i+1}^{+}=h_{i}^{+}+n_{i}\hat{r}.$$

**Figure 4:**
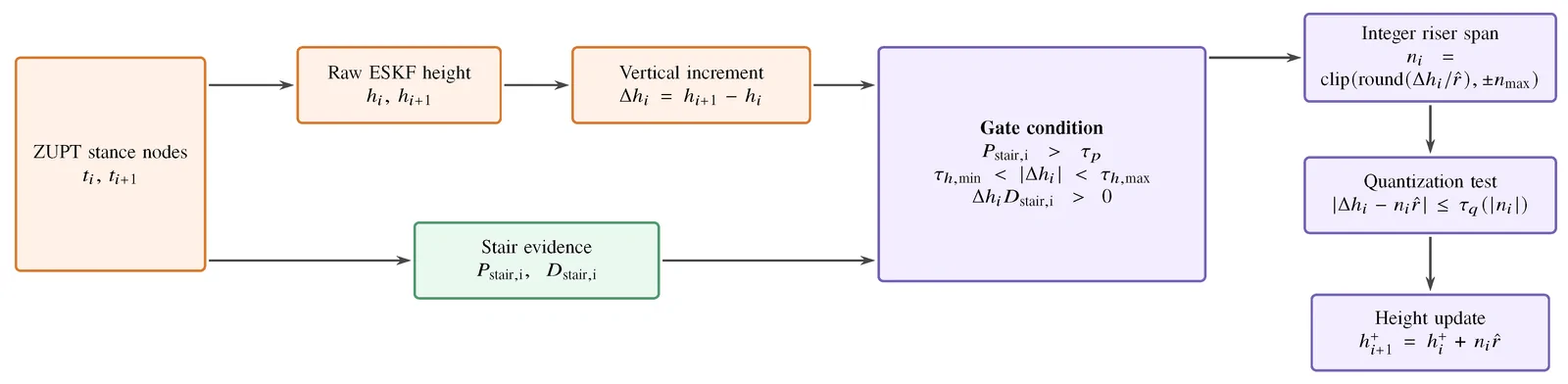
Probability-gated integer-riser update. The revised diagram explicitly shows the probability, magnitude, directional-consistency, and quantization-residual tests used by Equations (29)–(32).

Between stance nodes, the raw inertial height is smoothly adjusted to pass through the constrained nodes. Figure 4 summarizes the decision.

### 3.7. Algorithms and Implementation Notes

Algorithm 1 summarizes the online process. Feature extraction runs when a new window is available; mechanization and ESKF propagation run at the IMU rate; heading and stair updates run only at eligible nodes. All thresholds and covariance limits were fixed on development sequences before evaluation. The literature baselines use the same raw IMU and common ESKF where applicable and are reported as paper-guided adaptations.
**Algorithm 1:** Probability-driven adaptive foot-mounted INS
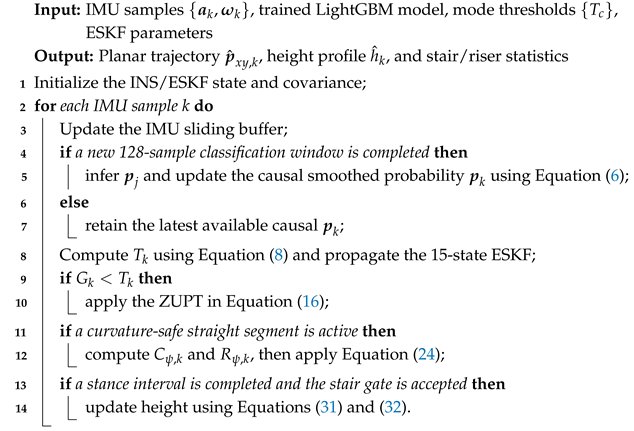


## 4. Experiments and Results

### 4.1. Experimental Setup

The evaluation follows the information flow of the proposed system and contains four experiments: motion classification, training and detector diagnostics, closed rectangular planar navigation, and multi-level stair-height reconstruction. This order first establishes whether the probability front-end is accurate and calibrated, then checks whether the learned probabilities produce physically plausible detector behavior, and finally evaluates the planar and vertical navigation branches. All data collection and physical navigation experiments were conducted in outdoor pedestrian environments. During trajectory estimation, no GNSS measurements, map constraints, scene images, known endpoints, or route geometry were supplied to the navigation filter; the photographed scenes, route markings, and geometric references were used only for documentation and performance evaluation.

**Figure 5:**
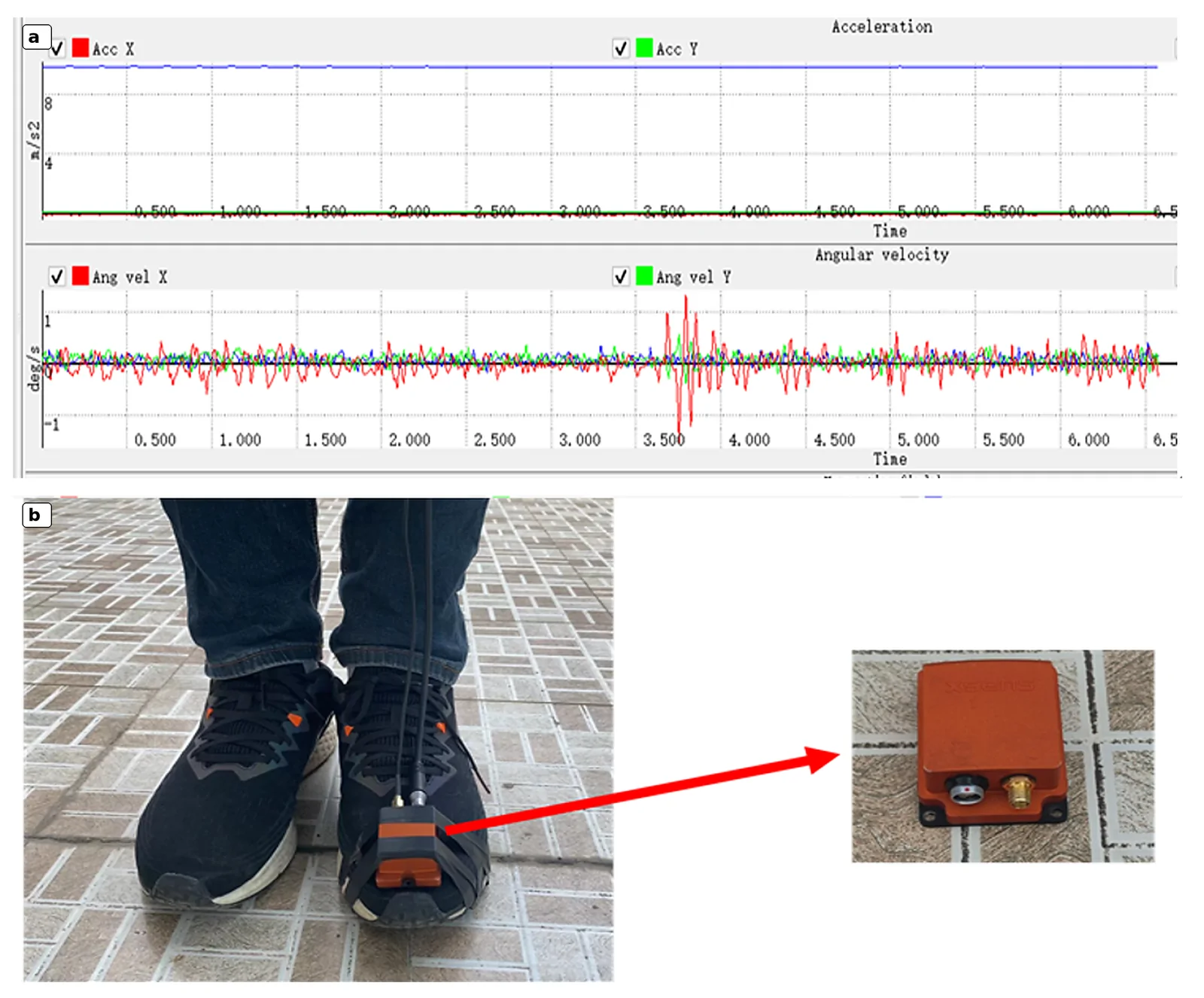
Experimental platform based on the foot-mounted Xsens MTi-G-710. The (**a**) shows representative acceleration and angular-rate recordings (red, green, and blue traces denote the x-, y-, and z-axis components, respectively), and the (**b**) shows the sensor installation on the shoe and the IMU unit.

The experimental platform is shown in Figure 5. An Xsens MTi-G-710 inertial measurement unit (Xsens Technologies B.V., Enschede, The Netherlands) was fixed on the instep of the participant’s shoe to acquire synchronized tri-axial acceleration and angular-rate measurements. All navigation sequences were sampled and processed at 100.00 Hz. The representative monitoring interface and the foot-mounted installation are shown together to document both the recorded inertial signals and the sensor placement. The main accelerometer and gyroscope specifications used in the experiments are summarized in Table 1.

Classification windows contain 128 samples with a stride of 64. Every classifier uses the same held-out-subject split and per-subject normalization computed from each subject’s complete unlabeled window collection. The protocol is therefore transductive with respect to normalization rather than a strict cold-start stream. Accuracy and macro-F1 quantify hard-label recognition, whereas NLL and the Brier score measure probability quality because the navigation filter consumes probabilities rather than only class labels. All classification baselines receive exactly the same windows and labels.

**Figure 6:**
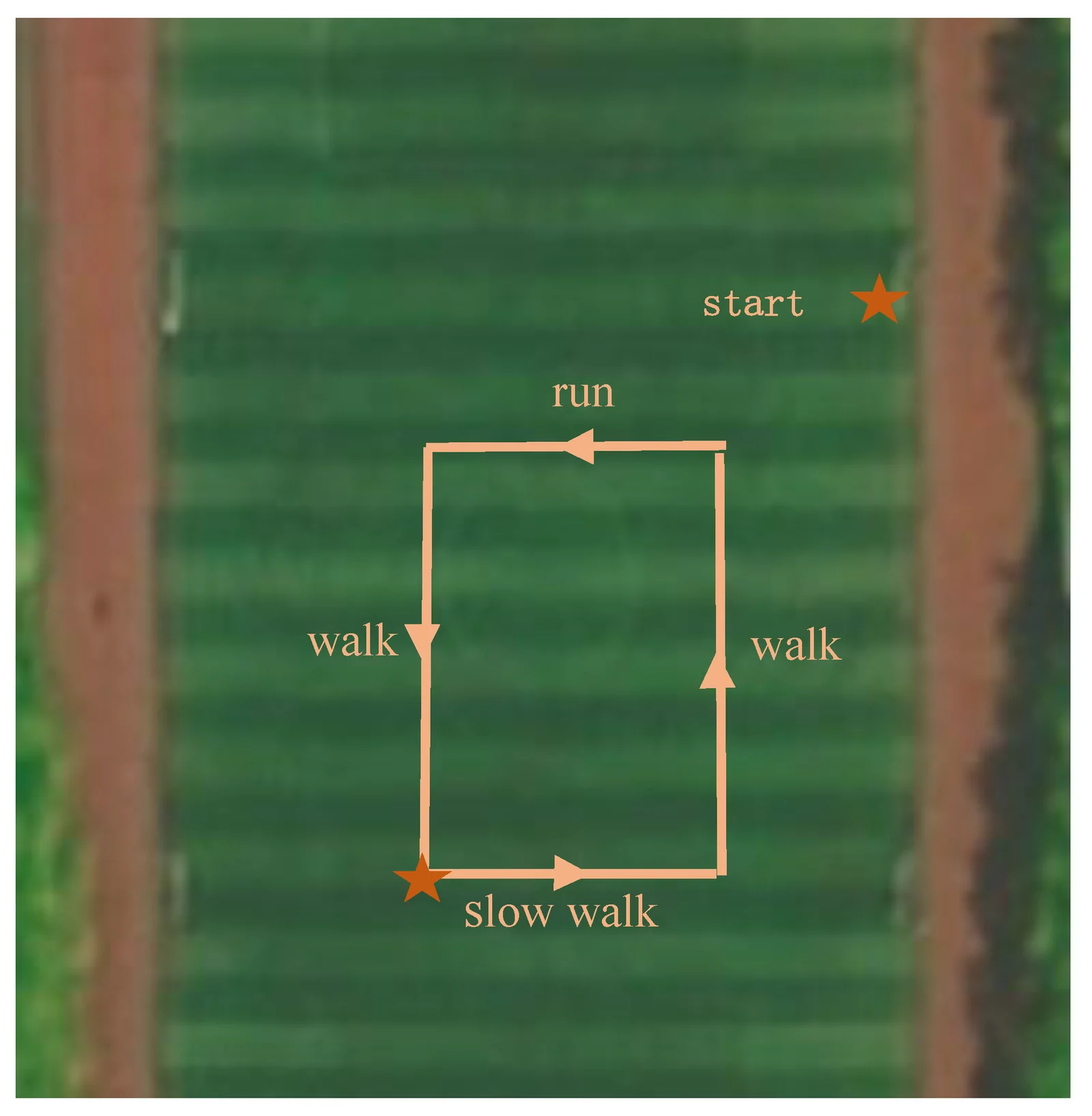
Outdoor closed rectangular planar-navigation test scene. The arrows indicate the traversal direction, and the marked points indicate the start and end of the experimental sequence.

The outdoor closed rectangular test scene is shown in Figure 6. The route was traversed according to the indicated direction and contains successive slow-walking, normal-walking, running, and normal-walking portions. The reported closure error is computed from the estimated start and end positions.

**Figure 7:**
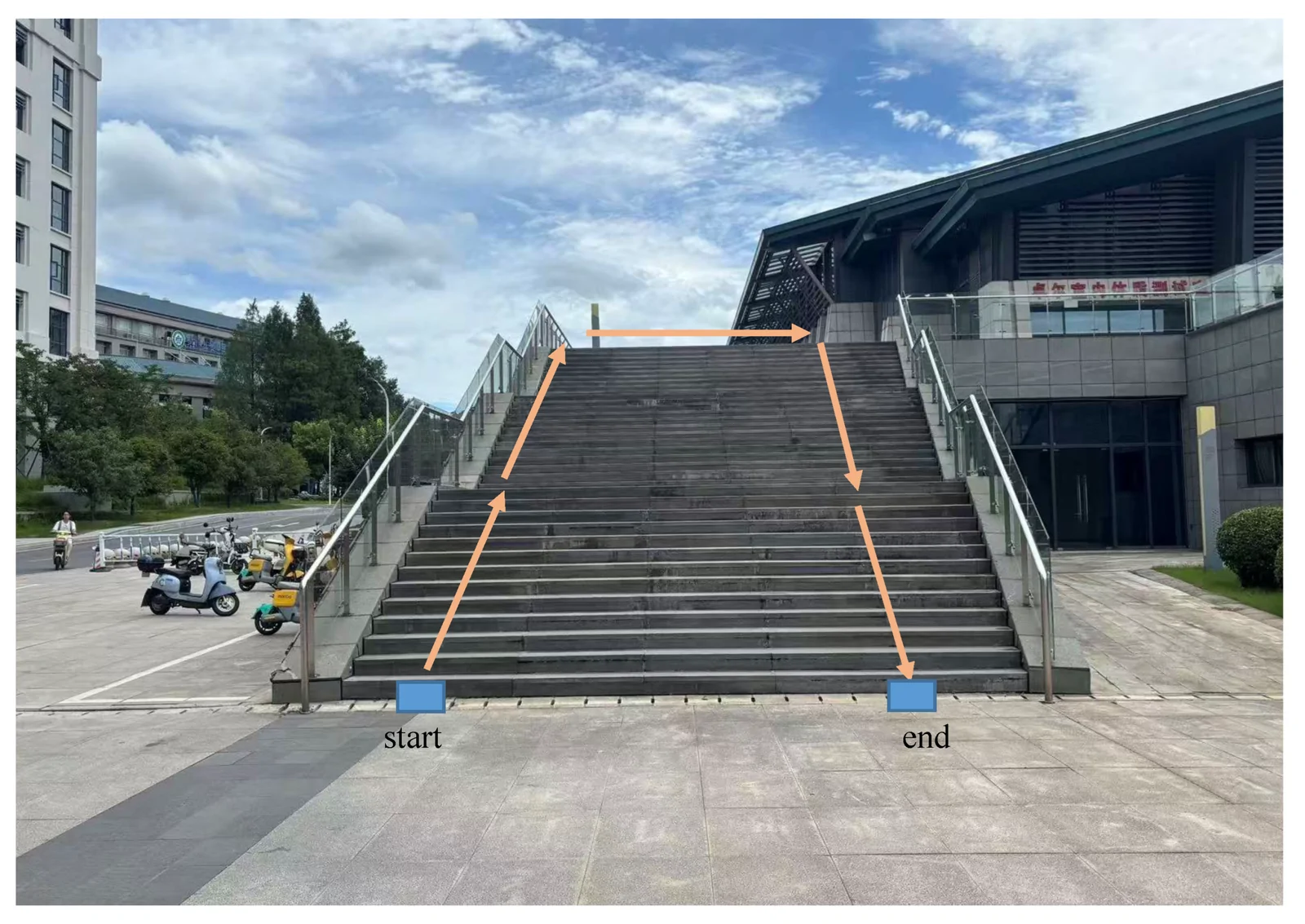
Outdoor multi-level stair-height reconstruction test scene. The arrows show the ascent, upper-landing transition, and descent from the marked start point to the endpoint. The non-English text visible on the building is incidental background signage and is unrelated to the experiment.

The outdoor multi-level stair test scene is shown in Figure 7. The participant started at the lower left, ascended the left side of the staircase, crossed the upper landing, and descended along the right side to the marked endpoint. The complete route contains 38 ascending and 38 descending risers, with a 0.16 m grid reference used only for evaluation. As in the planar experiment, the scene image and marked route are not used as filter inputs.

For fairness, all planar-navigation methods use the same raw IMU sequence, initial navigation state, 15-state ESKF mechanization, process-noise settings, and evaluation reference. The literature methods are paper-guided adaptations in which only the method-specific detector or heading observation is changed; no method receives the photographed scene, known endpoint, rectangular geometry, or reference trajectory. Table 2 summarizes the implementation roles.

The stair baselines are treated analogously: Raw ESKF contains no explicit vertical constraint, whereas Skog-JMM [15], Wang-Impact [38], and Moghaddam-ZHC [39] retain their method-specific vertical observation logic while using the same raw IMU and stance-node sequence where applicable. All tunable thresholds used in the proposed method and the paper-guided adaptations are fixed before evaluation rather than fitted to the reported endpoint or stair profile.

### 4.2. Experiment 1: Motion Classification

Table 3 shows that MF-LightGBM reaches 96.24% accuracy and 96.17% macro-F1. Compared with MobileHARC [27], the strongest deep baseline, the gains are 5.38 and 4.47 percentage points. NLL falls from 0.34 to 0.13, and the Brier score falls from 0.16 to 0.06, corresponding to reductions of 63.42% and 61.83%. These probability-quality gains are operationally important: a classifier that is accurate but overconfident can assign an unrealistically small pseudo-measurement covariance and thereby inject a larger navigation error than a cautious classifier.

The advantage is not obtained by increasing model size. MF-LightGBM uses 80 shallow trees, whereas the neural baselines range from approximately 16.58 thousand to 1.08 million trainable parameters. The explicit features expose cadence, harmonic energy, impact sharpness, and cross-axis coupling directly, reducing the amount of representation learning required from a moderate-size dataset. MobileHARC [27] remains the strongest neural baseline because its compact attention blocks preserve both local impacts and longer temporal context, but it obtains poorer NLL and Brier scores. HART [26] has strong running and upstairs recognition yet loses robustness on static and downstairs samples, suggesting that its sensor-wise attention is sensitive to the limited training diversity of these classes. DeepConvLSTM [25] models recurrent temporal context well but confuses downstairs with level walking, while LIMUNet [28]’s aggressive compression removes part of the class-specific detail needed to separate similar stance–swing patterns.

**Figure 8:**
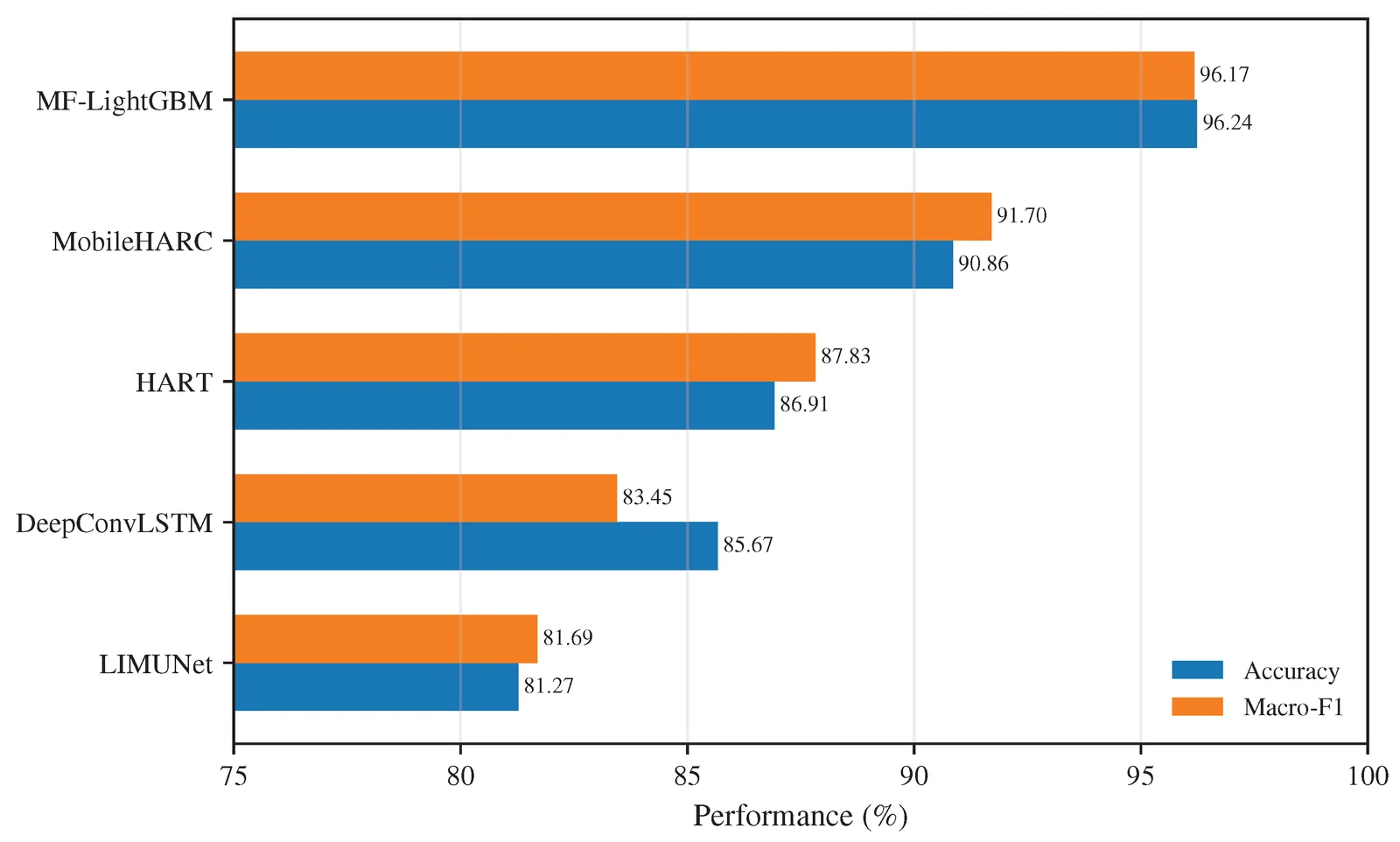
Classification comparison under the shared window protocol. The figure emphasizes hard-label performance, while NLL and Brier scores are reported in Table 3.

Figure 8 summarizes the overall classification accuracy and macro-F1 comparison.

**Figure 9:**
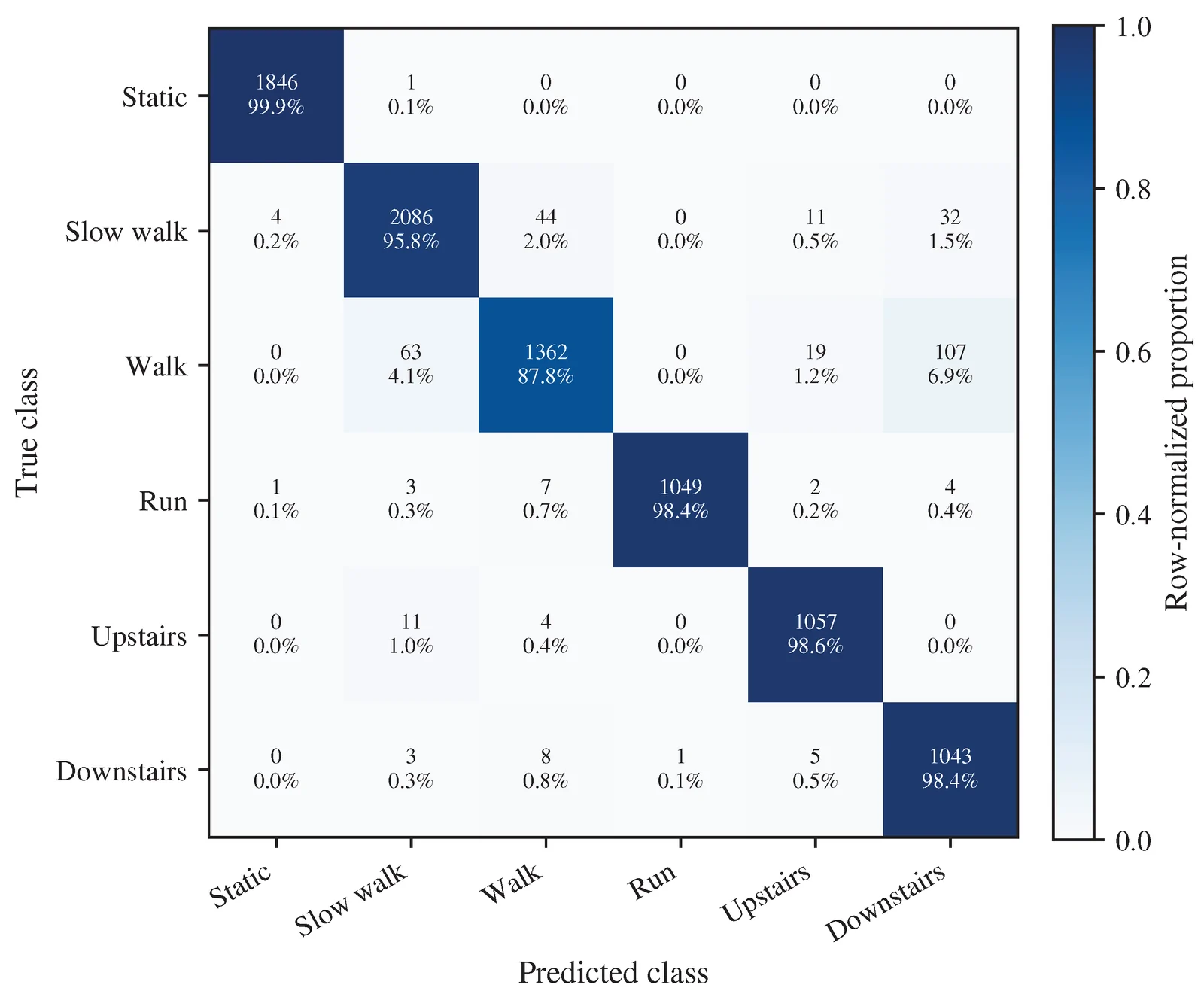
Confusion matrix of MF-LightGBM. The dominant residual ambiguity is among slow walking, walking, and downstairs rather than between static and high-dynamic motion.

Table 4 and Figure 9 localize the residual errors. Static and running are highly distinctive because they have either near-zero dynamics or strong cadence and impact energy. Most remaining ambiguity occurs among slow walking, walking, and downstairs. These activities share similar periodicity and can differ mainly in vertical impulse distribution and foot orientation, which are more subject-dependent. The proposed navigation modules do not trust a single hard decision in this region: stair evidence uses the sum of upstairs and downstairs probabilities together with vertical inertial change, and the SHOE threshold is a convex mixture of all six class probabilities.

### 4.3. Experiment 2: Training Behavior and Adaptive-Detector Diagnostics

**Figure 10:**
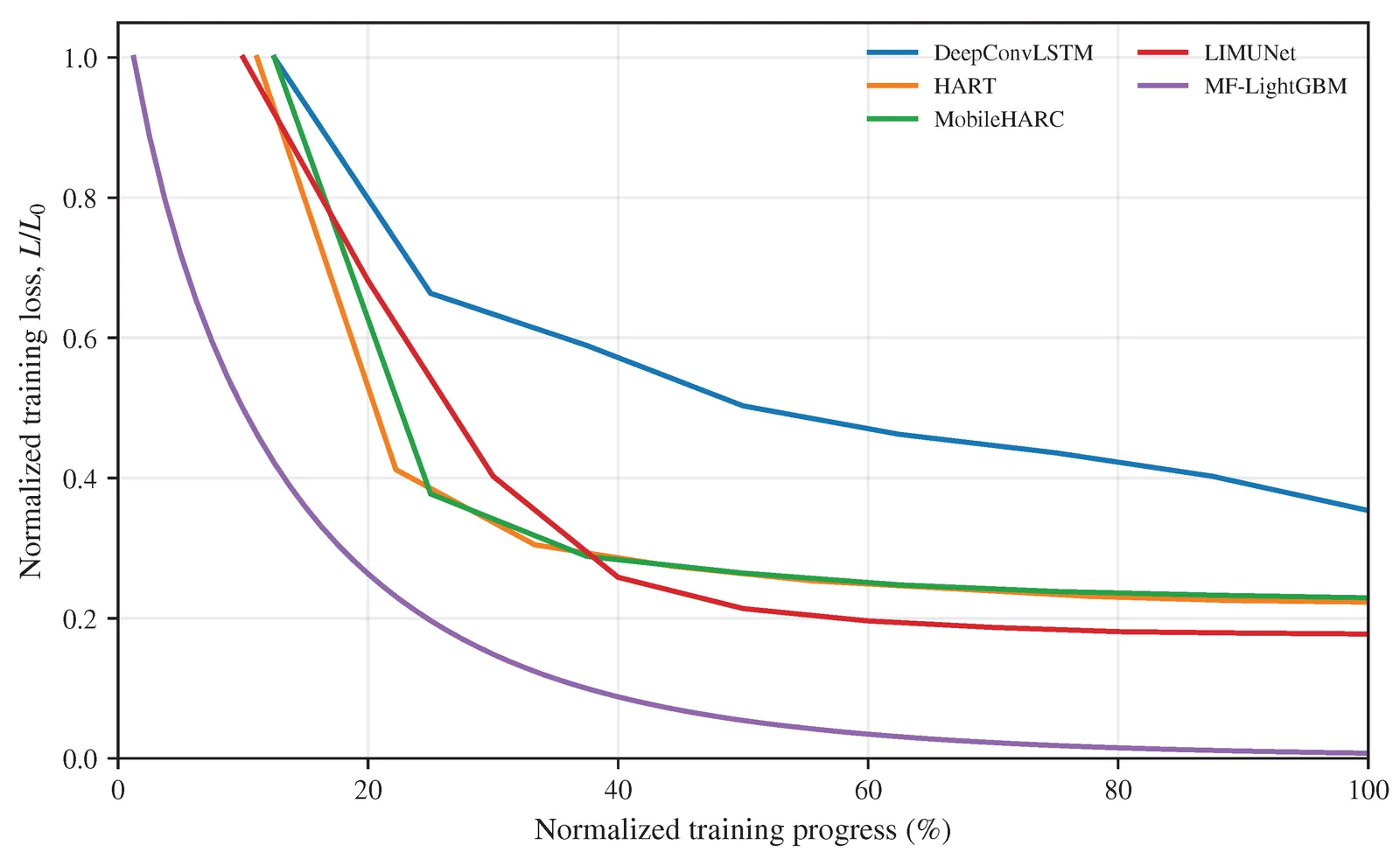
Normalized training-loss histories. The curves diagnose convergence only; final model selection uses the held-out metrics in Table 3.

Figure 10 provides an optimization diagnostic before navigation is evaluated. The losses are normalized by their initial values because the tree ensemble and neural networks do not optimize identical objectives, so the curves should not be interpreted as directly comparable objective values. MF-LightGBM reaches a low normalized loss rapidly, which is consistent with the use of explicit gait descriptors. MobileHARC shows smooth neural optimization and is also the strongest deep baseline. LIMUNet likewise converges without an obvious optimization failure, while DeepConvLSTM shows a slower normalized loss decrease. These curves are therefore used only to identify gross optimization instability; they do not establish a causal explanation for the final accuracy differences among fundamentally different model classes.

Detector diagnostics then test whether the probability front-end improves the physical events used by the ESKF. The six-mode threshold mixture detects 31 running stance intervals and limits the maximum displacement between adjacent running stances to approximately 1.27 m. Fixed-threshold processing detects too few running contacts and yields a nearly 40.00 m third side. The probability-adaptive detector reduces that side to 33.29 m without using the known side length. This change is consistent with recovered stance events, not endpoint fitting. Slow walking retains a lower threshold, whereas normal walking and running receive higher effective thresholds according to their probability mixtures. Consequently, the detector changes smoothly at gait transitions instead of producing abrupt mode-switch artifacts.

### 4.4. Experiment 3: Closed Rectangular Planar Navigation

The endpoint metrics are

(33) $$e_{\mathrm{cl}}={∥{\hat{p}}_{N}-{\hat{p}}_{0}∥}_{2},$$

and

(34) $$e_{\mathrm{rel}}=\frac{e_{\mathrm{cl}}}{L_{\mathrm{ref}}}\times 100\%,$$

where $L_{\mathrm{ref}}$ is the same independently measured route length for every method and is used only for evaluation. Using each method’s estimated path length in the denominator would make the metric method-dependent and can reward trajectories that overestimate distance. Table 5 reports the closure metrics for all planar-navigation methods. Because different side errors can cancel at the endpoint, Table 6 also reports side lengths and opposite-side consistency. The comparison includes two velocity-only methods, SHOE-ESKF [2,3] and Wang-AWGF [12], and two methods with relative heading-drift correction, Guo-SIHDC [17] and Deng-HDR [16]. This grouping makes the source of improvement easier to interpret than a single ranked list.

The proposed method obtains a 1.09 m closure error and a 0.68% relative error. This is 51.01% lower than Deng-HDR, 92.93% lower than Guo-SIHDC, and 95.95% lower than fixed SHOE-ESKF. SHOE-ESKF is weakest because a fixed threshold misses high-dynamic running contacts and the remaining ZUPTs do not directly observe yaw. Wang-AWGF adapts contact detection but still lacks a heading-drift constraint, so improved stance timing alone cannot prevent cross-track accumulation. Guo-SIHDC applies interval heading-difference correction and recovers realistic side lengths, but its interval decisions are more sensitive to mixed-gait transitions and short local changes. Deng-HDR is the strongest literature baseline because the route contains long straight segments, yet its fixed correction strength treats clear and uncertain straight segments similarly.

**Figure 11:**
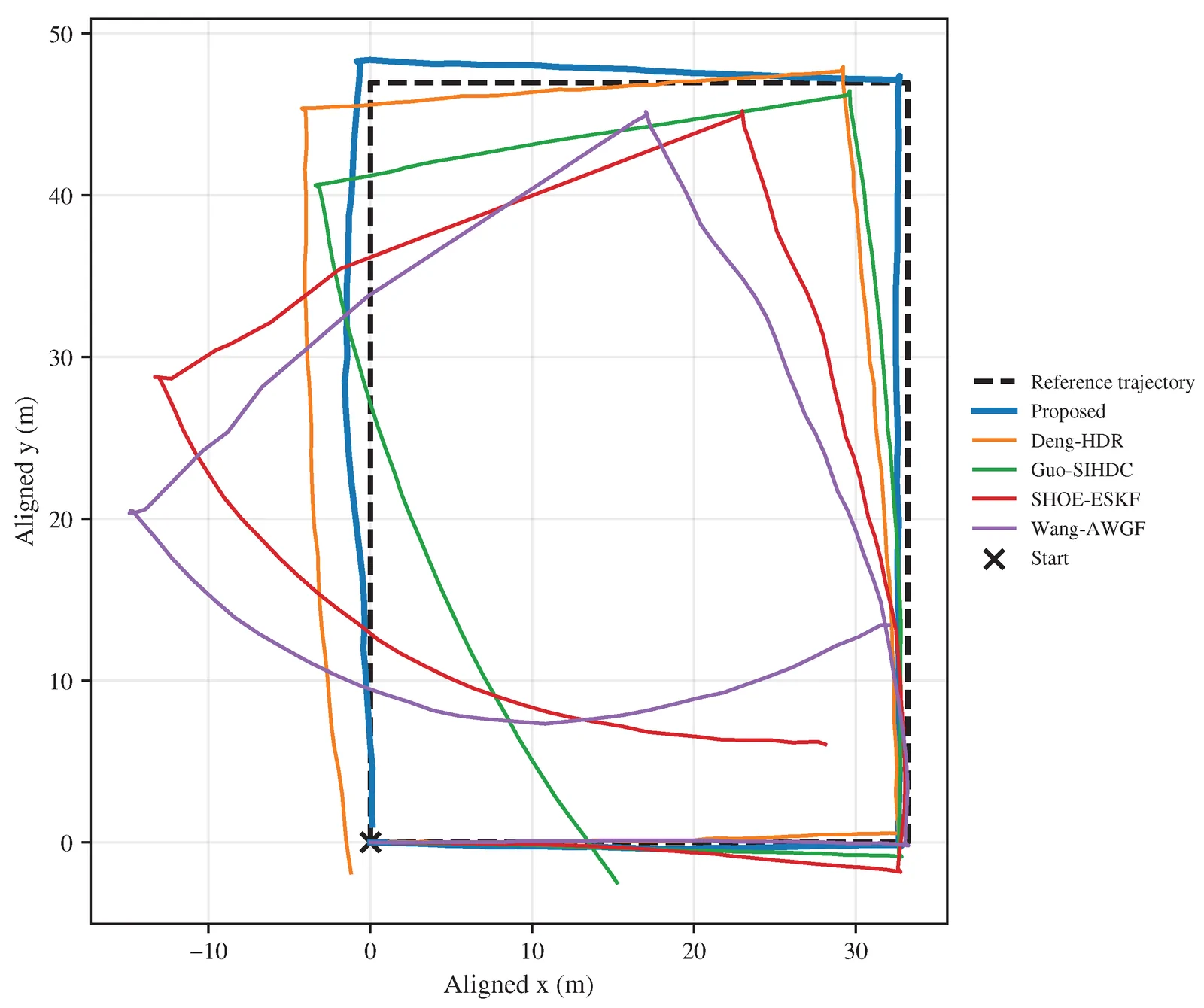
Closed rectangular trajectories. The dashed reference trajectory is used only for visual shape comparison and is not supplied to any filter.

Figure 11 shows the corresponding closed-rectangle trajectories.

**Figure 12:**
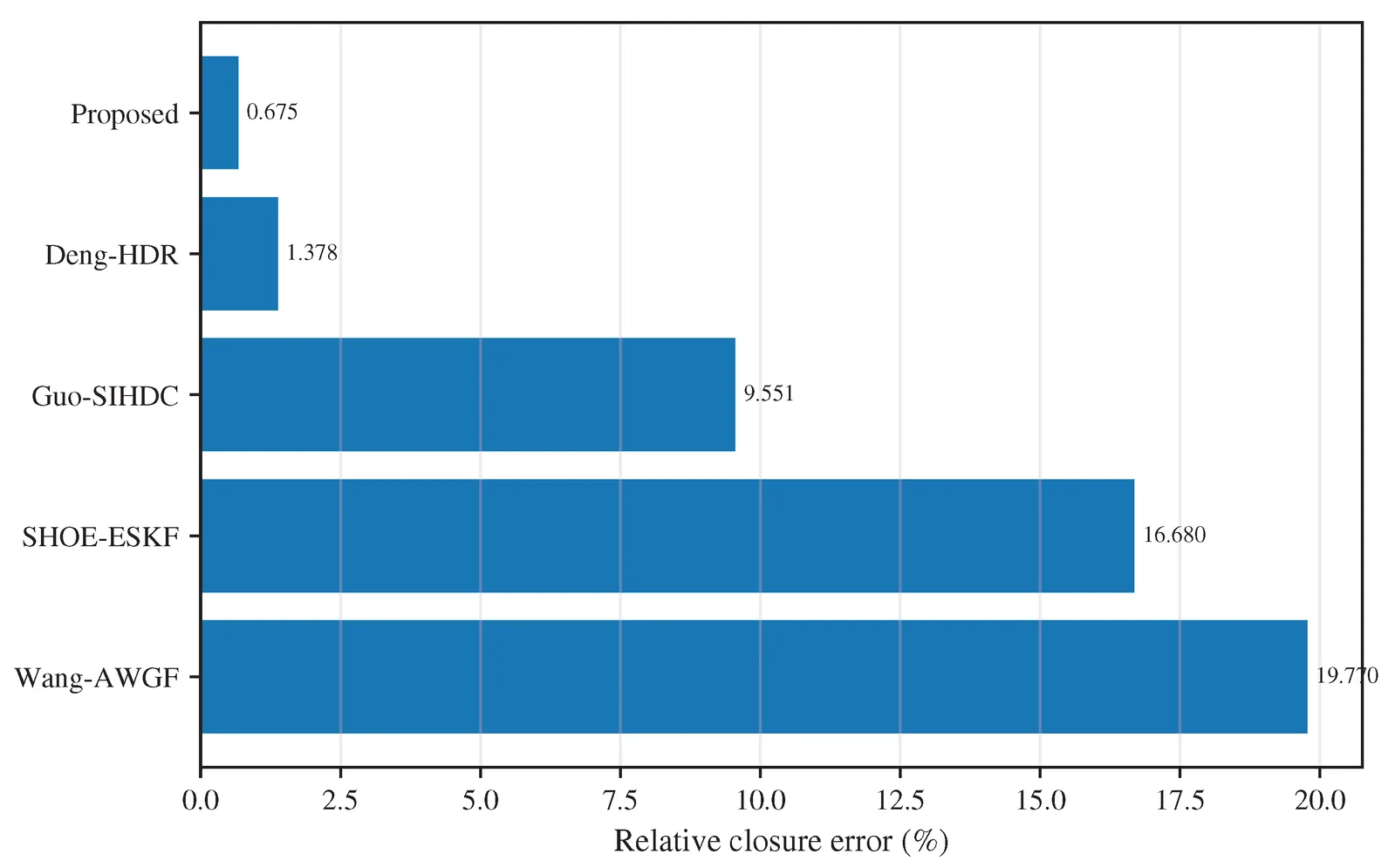
Relative closure error. The proposed method reduces the error by 51.01% relative to the strongest literature baseline, Deng-HDR.

The side statistics confirm that the small endpoint error is not a cancellation artifact. The proposed short sides differ by 0.17 m and the long sides differ by 0.89 m. SHOE-ESKF and Wang-AWGF overestimate the running side by more than 6.00 m, which is consistent with missed stance events rather than a pure heading error. Guo-SIHDC and Deng-HDR recover side scale but still accumulate orientation error over successive turns. MA-LSHC improves on Deng-HDR because curvature gates reject persistent turning, while the covariance in Equation (26) weakens updates when straightness, gait suitability, or classifier confidence is low. Strong updates are therefore concentrated on locally reliable straight segments. Figure 12 summarizes the corresponding relative closure errors.

### 4.5. Experiment 4: Multi-Level Stair-Height Reconstruction

Table 7 summarizes the stance-node height errors for the stair experiment. The proposed method achieves a RMSE of 0.13 m, a MAE of 0.11 m, and a maximum node error of 0.21 m. Its RMSE is 26.42% lower than Skog-JMM [15], 33.06% lower than Wang-Impact [38], 39.03% lower than Moghaddam-ZHC [39], and 60.93% lower than Raw ESKF. Unlike the planar closure metric, the stair errors are computed at stance nodes against the 0.16 m riser grid, so the comparison evaluates both local height accuracy and long-run ascent–descent consistency.

Table 8 reports the riser counts, transitions, and top-landing height statistics.

**Figure 13:**
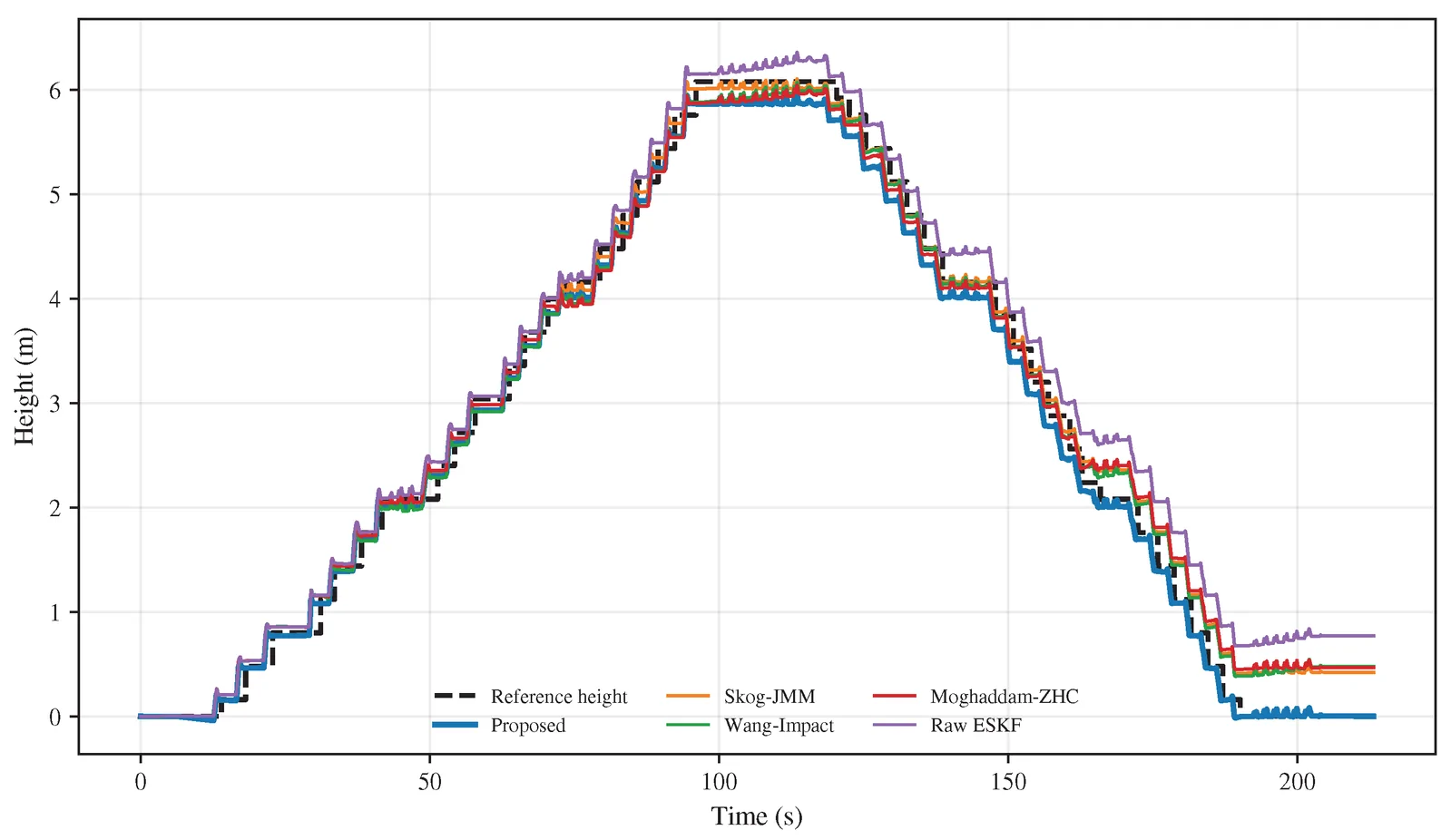
Height trajectories. The proposed method preserves the riser sequence and removes the terminal offset after descent.

Figure 13 visualizes the corresponding height profiles. Raw ESKF suffers from double-integrated vertical error because neither the ZUPT nor the inertial mechanization alone provides an absolute height reference. Wang-Impact suppresses part of the impulse-related stride error but does not impose long-run riser consistency, so a terminal offset remains. Moghaddam-ZHC is effective for level-foot phases but relaxes its constraint on stairs, allowing residual drift and missing one riser in each direction. Skog-JMM has the smallest top-landing error because its switching vertical lock is locally effective near the landing; however, it finishes with a 0.42 m residual after the complete ascent–descent sequence.

**Figure 14:**
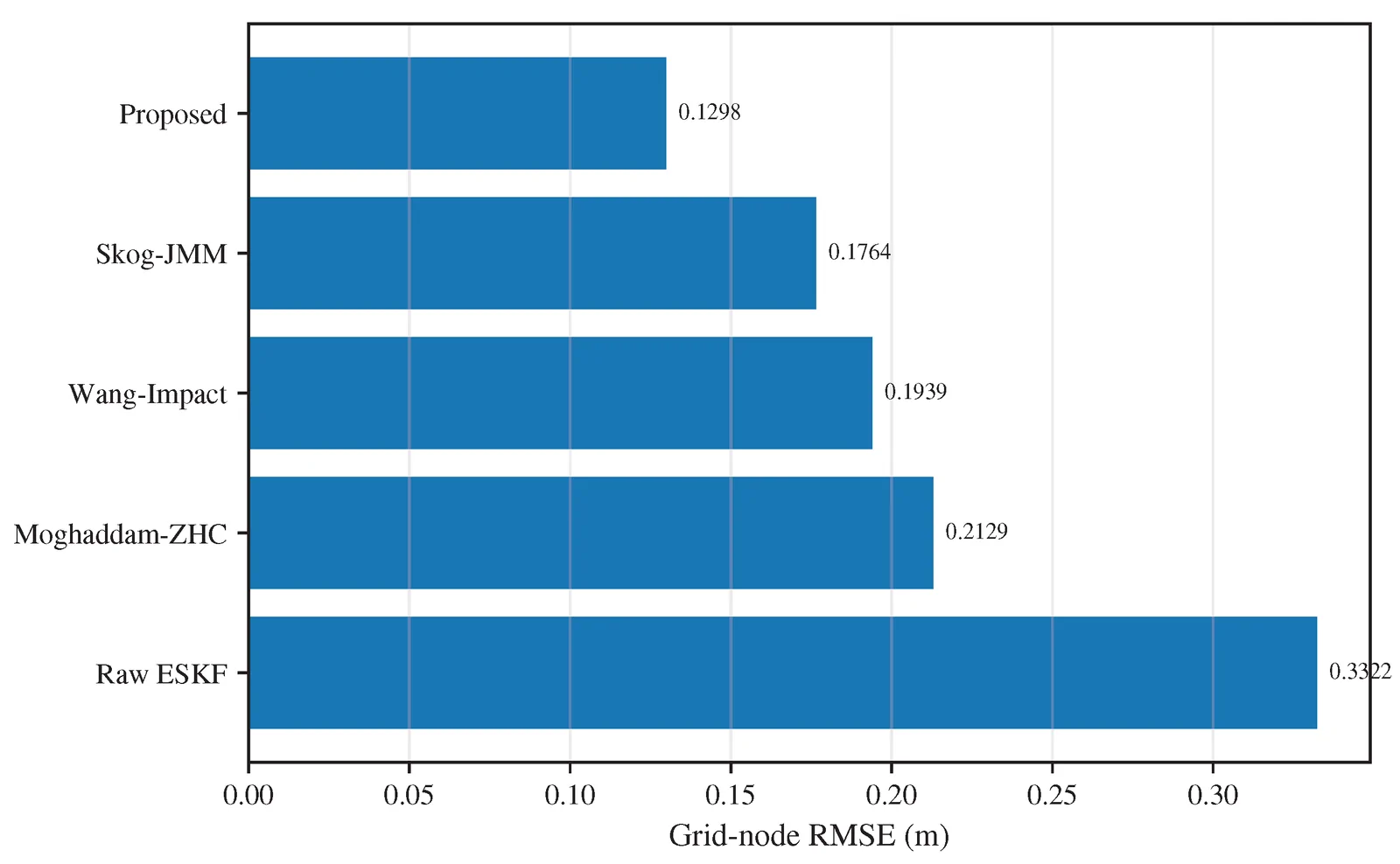
Grid-node RMSE. Probability gating and integer-riser consistency reduce the proposed RMSE to 0.13 m.

Figure 14 compares the grid-node RMSE across the evaluated stair-height methods.

## 5. Discussion

The results show that the six-mode probability vector is most useful as a shared reliability cue rather than as a hard activity label. It adapts stance detection, weakens or strengthens the local heading pseudo-measurement, and gates stair-height quantization while leaving the physical ESKF unchanged. This common interface explains the gains observed in both the planar and vertical experiments. The online input is only a synchronized six-axis foot-mounted IMU; training is performed offline. In the CPU-only timing check, the complete MF-LightGBM front-end required 5.99 ms per 128-sample window; under the same setting, DeepConvLSTM, HART, MobileHARC, and LIMUNet-Tiny required 0.66, 2.57, 1.15, and 0.66 ms per window, respectively. With a 64-sample stride at 100 Hz, probabilities are updated every 0.64 s, leaving ample real-time margin.

The current scope nevertheless has clear limitations. Per-subject unlabeled normalization makes the held-out-subject protocol transductive rather than a strict cold-start test. MA-LSHC constrains local heading change but cannot observe the global yaw offset and requires occasional locally straight motion. The stair update also assumes sufficiently regular riser geometry within prescribed physical bounds. These limitations define the present operating envelope and motivate evaluation in more diverse indoor and GNSS-challenged environments.

## 6. Conclusions

This paper presented a probability-driven adaptive foot-mounted inertial navigation framework for infrastructure-free pedestrian positioning in GNSS-denied or GNSS-degraded environments. A motion-feature LightGBM front-end outputs six-mode probabilities, which are used to adapt the SHOE threshold, control local heading pseudo-measurement covariance, and gate integer-riser height updates. The classifier achieved 96.24% accuracy. In an outdoor mixed-gait closed rectangular route, the proposed method reduced the relative closure error to 0.68%, outperforming both the adaptive ZUPT and heading-drift baselines. In the outdoor stair experiment, the method achieved a 0.13 m grid-node RMSE and identified 38 ascending and 38 descending risers. These findings show that motion probabilities can serve as continuous reliability variables inside a foot-mounted inertial filter under controlled outdoor mixed-gait and multi-level conditions. Further work will evaluate the method in complex indoor spaces and other diverse GNSS-challenged environments.

## Figures and Tables

**Table 1:** Main inertial-sensor specifications of the MTi-G-710 used in the experiments.

Sensor	Parameter	Value
Accelerometer	Measurement range	18 g
	Noise density	$80\;\mu g/\sqrt{\mathrm{Hz}}$
	Bias stability	40μg
Gyroscope	Measurement range	$\pm 1000^{\circ }/s$
	Noise density	$0.01^{\circ }/s/\sqrt{\mathrm{Hz}}$
	Bias stability	$10^{\circ }/h$

**Table 2:** Implementation of the planar-navigation baselines under the common comparison protocol.

Method	Implementation Under the Common Protocol
SHOE-ESKF [2,3]	Fixed SHOE threshold for stance detection; common 15-state ESKF and ZUPT update; no heading-drift pseudo-measurement.
Wang-AWGF [12]	Paper-guided gait-frequency adaptation of the SHOE detection settings; common ESKF/ZUPT back-end; no additional heading constraint.
Guo-SIHDC [17]	Common ZUPT-aided ESKF augmented with motion-recognition-supported interval heading-difference correction; no map, endpoint, or route-geometry input.
Deng-HDR [16]	Common ESKF augmented with gait-classification-aided heading-drift reduction/zero-integrated-heading-rate logic following the published method; correction strength is not probability-adaptive.

**Table 3:** Overall classification performance under the fixed held-out-subject protocol with per-subject unlabeled normalization.

Model	Accuracy (%)	Macro-F1 (%)	Weighted-F1 (%)	NLL	Brier
MF-LightGBM	96.24	96.17	96.23	0.13	0.06
MobileHARC	90.86	91.70	90.86	0.34	0.16
HART	86.91	87.83	87.09	0.36	0.17
DeepConvLSTM	85.67	83.45	85.53	0.44	0.21
LIMUNet	81.27	81.69	80.77	0.52	0.24

**Table 4:** Per-class F1 scores of the compared classification methods.

Model	Static	Slow Walk	Walk	Run	Upstairs	Downstairs
MF-LightGBM	99.84	96.04	91.53	99.15	97.60	92.88
MobileHARC	99.46	84.88	79.59	98.77	98.28	89.22
HART	79.69	88.61	85.55	99.10	98.32	75.68
DeepConvLSTM	99.49	90.78	78.24	96.93	76.06	59.19
LIMUNet	69.60	83.21	81.19	88.56	78.75	88.86

**Table 5:** Closed-rectangle trajectory results. The closure error is the start–end distance after a full loop.

Method	Closure Error (m)	Path Length (m)	Relative Error (%)
Proposed	1.09	161.63	0.68
Deng-HDR	2.23	161.72	1.38
Guo-SIHDC	15.43	161.59	9.55
SHOE-ESKF	28.75	172.37	16.68
Wang-AWGF	34.74	175.71	19.77

**Table 6:** Side-length and shape statistics of the closed rectangular route. $S_{i}$ denotes the estimated length of side *i*; $\Delta_{s}$ and $\Delta_{l}$ are the absolute differences between the two short and two long sides, respectively.

Method	$S_{1}$ (m)	$S_{2}$ (m)	$S_{3}$ (m)	$S_{4}$ (m)	$\Delta_{s}$ (m)	$\Delta_{l}$ (m)
Proposed	33.12	46.52	33.29	47.41	0.17	0.89
Deng-HDR	33.15	46.49	33.28	47.51	0.13	1.03
Guo-SIHDC	33.11	46.45	33.25	47.49	0.14	1.04
SHOE-ESKF	33.10	47.52	39.98	50.50	6.89	2.98
Wang-AWGF	33.51	48.25	40.67	52.24	7.16	4.00

**Table 7:** Stair-height reconstruction errors at stance nodes. Landing statistics are reported separately in Table 8.

Method	RMSE (m)	MAE (m)	Max. Error (m)	Final Height (m)
Proposed	0.13	0.11	0.21	−0.00
Skog-JMM	0.18	0.12	0.44	0.42
Wang-Impact	0.19	0.15	0.48	0.45
Moghaddam-ZHC	0.21	0.16	0.48	0.46
Raw ESKF	0.33	0.23	0.77	0.75

**Table 8:** Stair detection and landing-height statistics.

Method	Up Risers	Down Risers	Transitions	Top Height (m)	Top Error (m)
Proposed	38.00	38.00	41.00	5.87	0.21
Skog-JMM	38.00	38.00	41.00	6.01	0.07
Wang-Impact	38.00	38.00	41.00	5.92	0.16
Moghaddam-ZHC	37.00	37.00	39.00	5.89	0.19
Raw ESKF	38.00	38.00	41.00	6.19	0.11

## Data Availability

The original contributions presented in this study are included in the article. Further inquiries can be directed to the corresponding author.
